# Supplementary material for: Continuously controllable photoconductance in freestanding BiFeO3 by the macroscopic flexoelectric effect
Source: Nat Commun. 2020 May 22;11:2571. doi: 10.1038/s41467-020-16465-5 (PMC7244550; doi:10.1038/s41467-020-16465-5)
Supplement: Supplementary file 1 — Supplementary Information [file 41467_2020_16465_MOESM1_ESM.pdf]

Supplementary Information

Continuously controllable photoconductance in free-standing $BiFeO_3$ by the macroscopic flexoelectric effect

Rui Guo[1,2a], Lu You[3a], Weinan Lin[1], Amr Abdelsamie[4], Xinyu Shu[1], Guowei Zhou[1], Shaohai Chen[1], Liang Liu[1], Xiaobing Yan[2*], Junling Wang[4,5*], Jingsheng Chen[1*]

1. Department of Materials Science and Engineering, National University of Singapore,117575, Singapore.
2. College of Electron and Information Engineering, Hebei University, Baoding 071002, China.
3. Jiangsu Key Laboratory of Thin Films, School of Physical Science and Technology, Soochow University, Suzhou 215006, China.
4. Department of Materials Science and Engineering, Nanyang Technological University, 639798, Singapore.
5. Department of physics, Southern University of Science and Technology, Shenzhen, China 518055.

[a] Equal contribution to this work

*Corresponding authors: msecj@nus.edu.sg; jwang@sustech.edu.cn; yanxiaobing@ime.ac.cn.

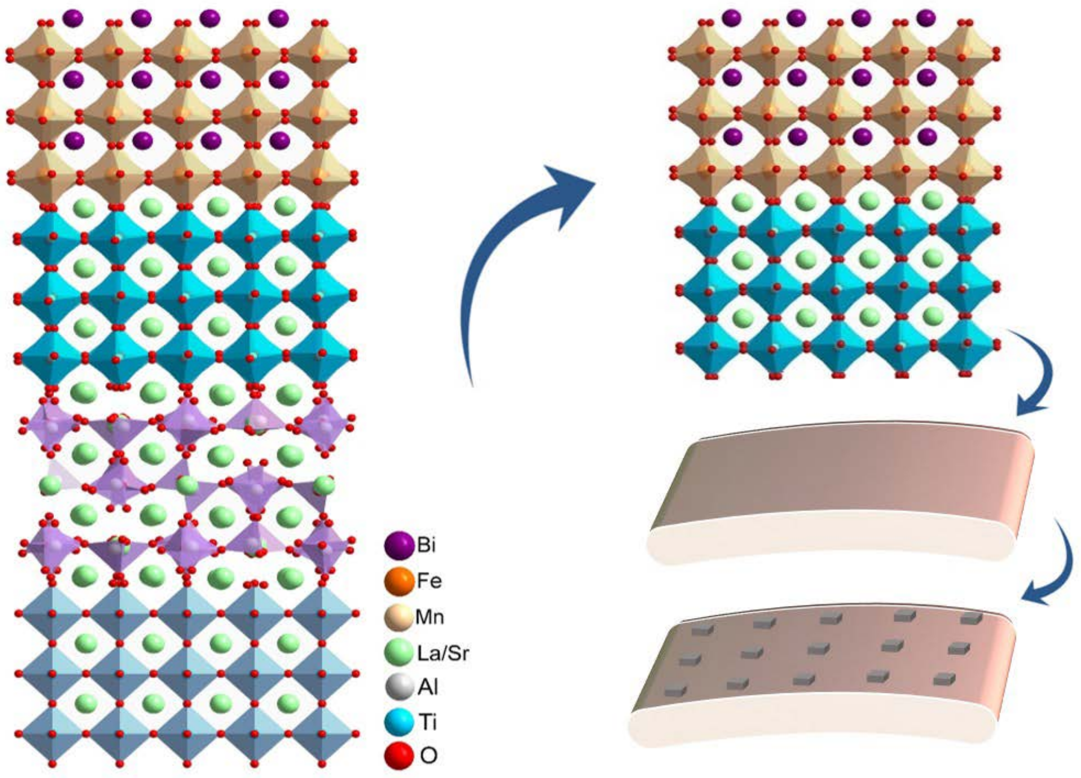

Supplementary Figure 1. Schematic of the thin film growth, transferring, and device patterning process.

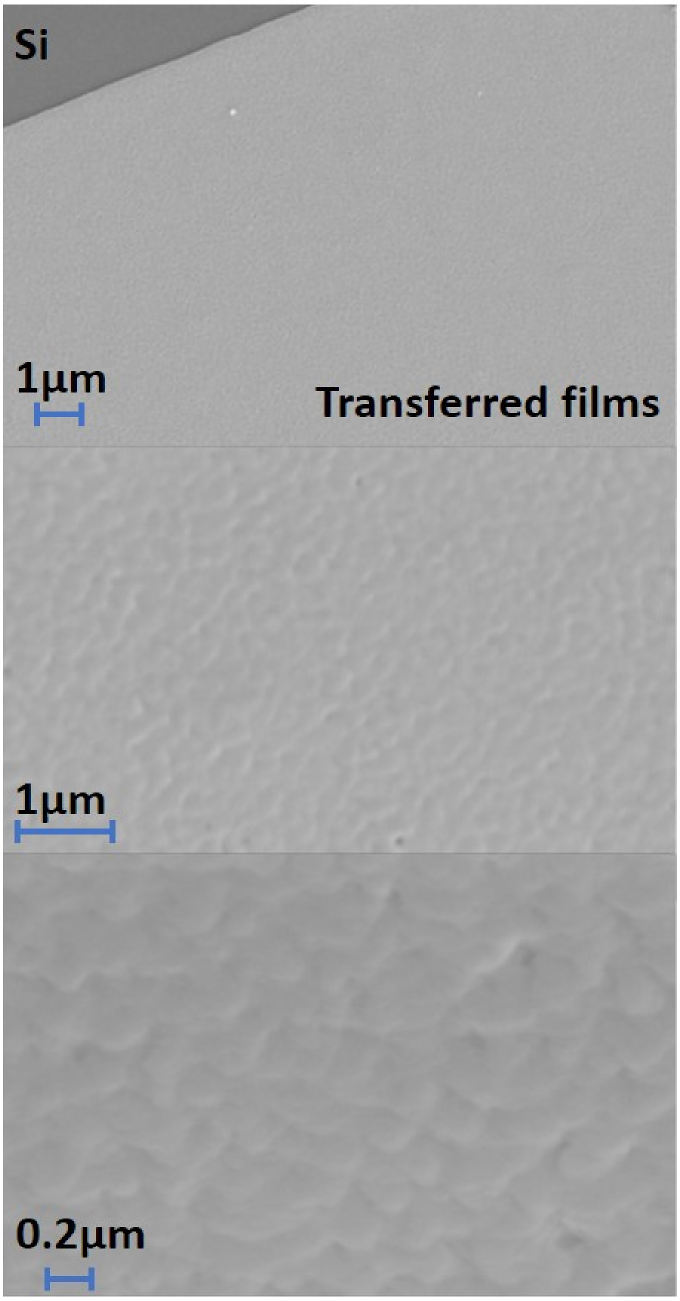

Supplementary Figure 2. Scanning electron microscopy (SEM) images. SEM images of transferred BFO/LSMO films on Si substrates with different scales.

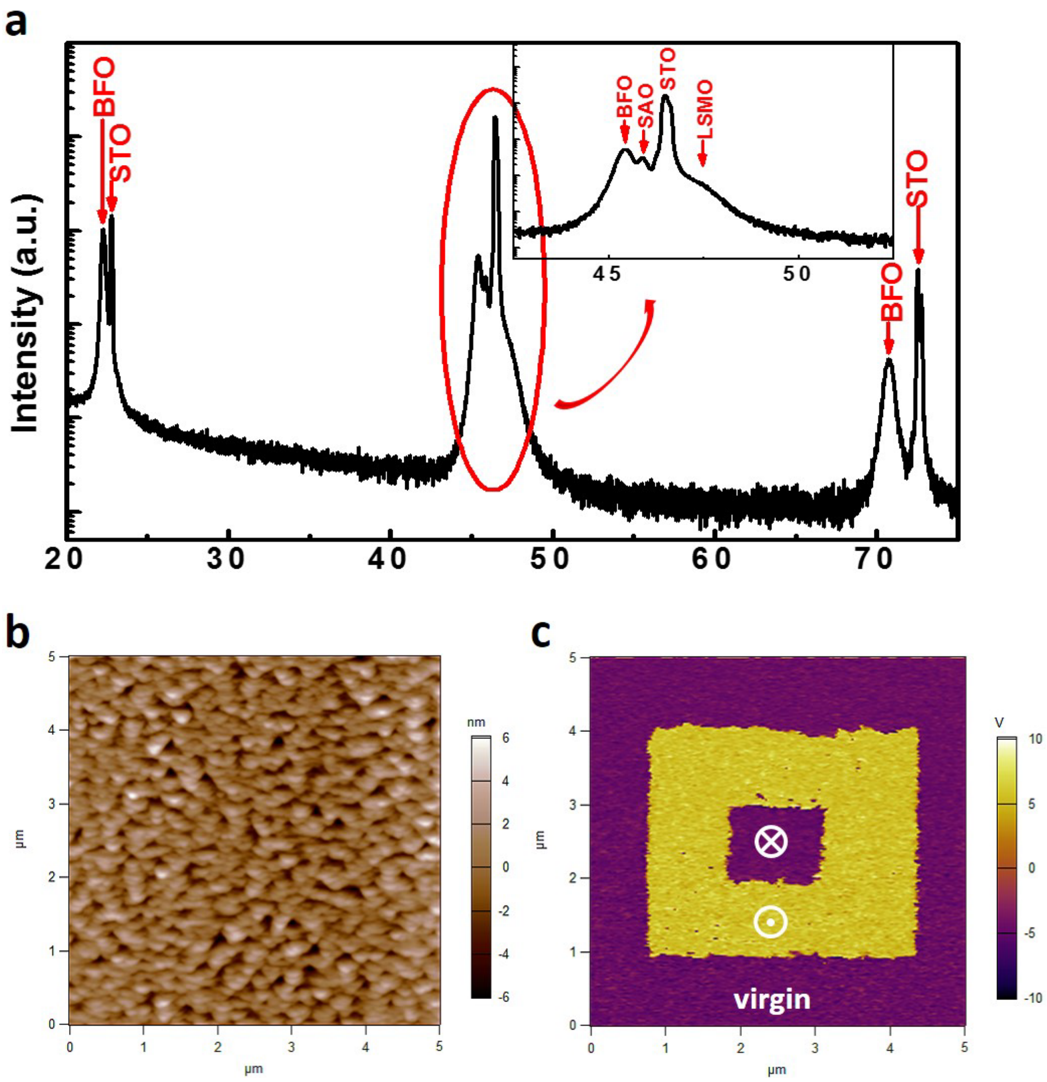

Supplementary Figure 3. XRD and PFM results. (**a**) XRD results of the as-grown BFO/LSMO/SAO/STO sample. (**b**) Topography of the freestanding BFO film. (**c**) In-plane PFM phase images of the freestanding BFO film, with the virgin state and the state after switching using a bias of -5 and +5 V, respectively. The inset shows the (002) peaks in the red circle.

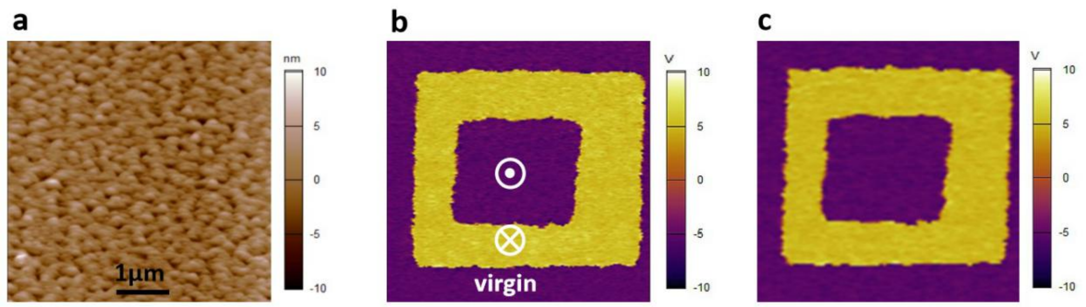

Supplementary Figure 4. PFM results of as-grown BFO/LSMO/SAO/STO sample. (**a**) Topography of as-grown BFO film. (**b**) Out-of-plane PFM image with the virgin state and the state after switching using a bias of -5 and +5 V, respectively. (**c**) The corresponding in-plane PFM image. The PFM results reveal that the as-grown BFO film has a virgin downward single domain structure. The freestanding BFO has the same polarization direction as the as-grown one with SAO layer.

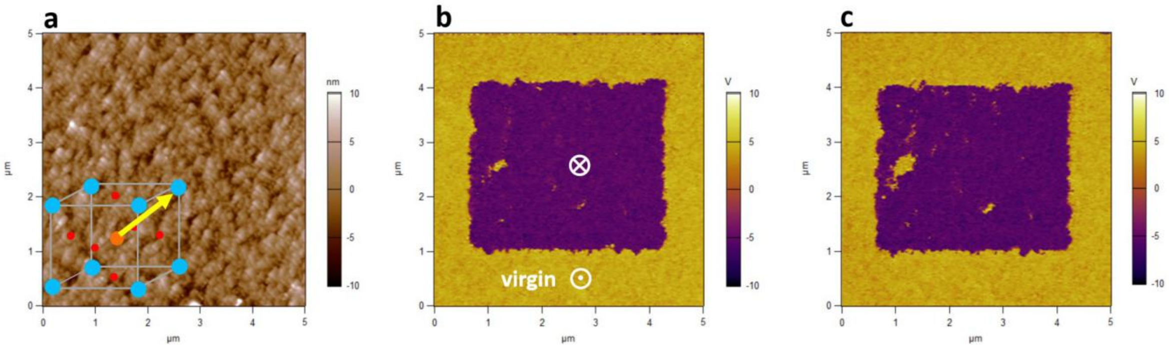

Supplementary Figure 5. PFM results of BFO film grown on STO without SAO. (**a**) The topography, (**b**) out-of-plane, and (**c**) in-plane PFM phase images of BFO film grown on STO without SAO layer. The insect of (**a**) shows the virgin polarization direction of BFO grown on STO with SAO.

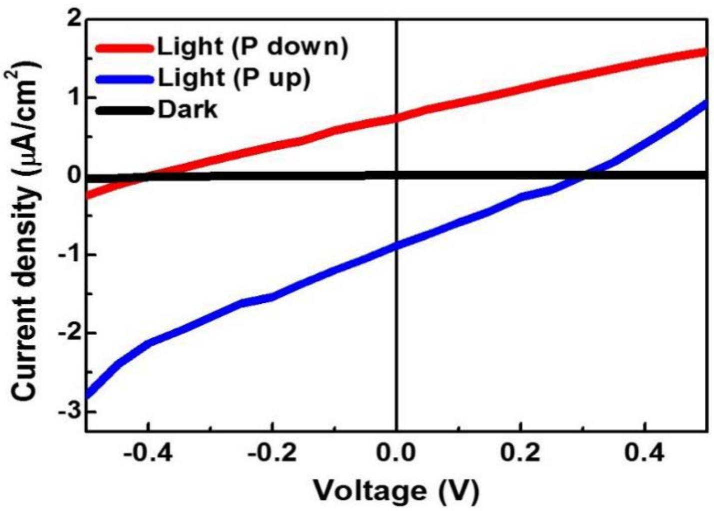

Supplementary Figure 6. Basic photovoltaic property of rigid Pt/BFO/LSMO/STO. *J-V* curves under light illumination (with light intensity of 20 mW/cm^2) and in dark for both polarizations of as grown sample.

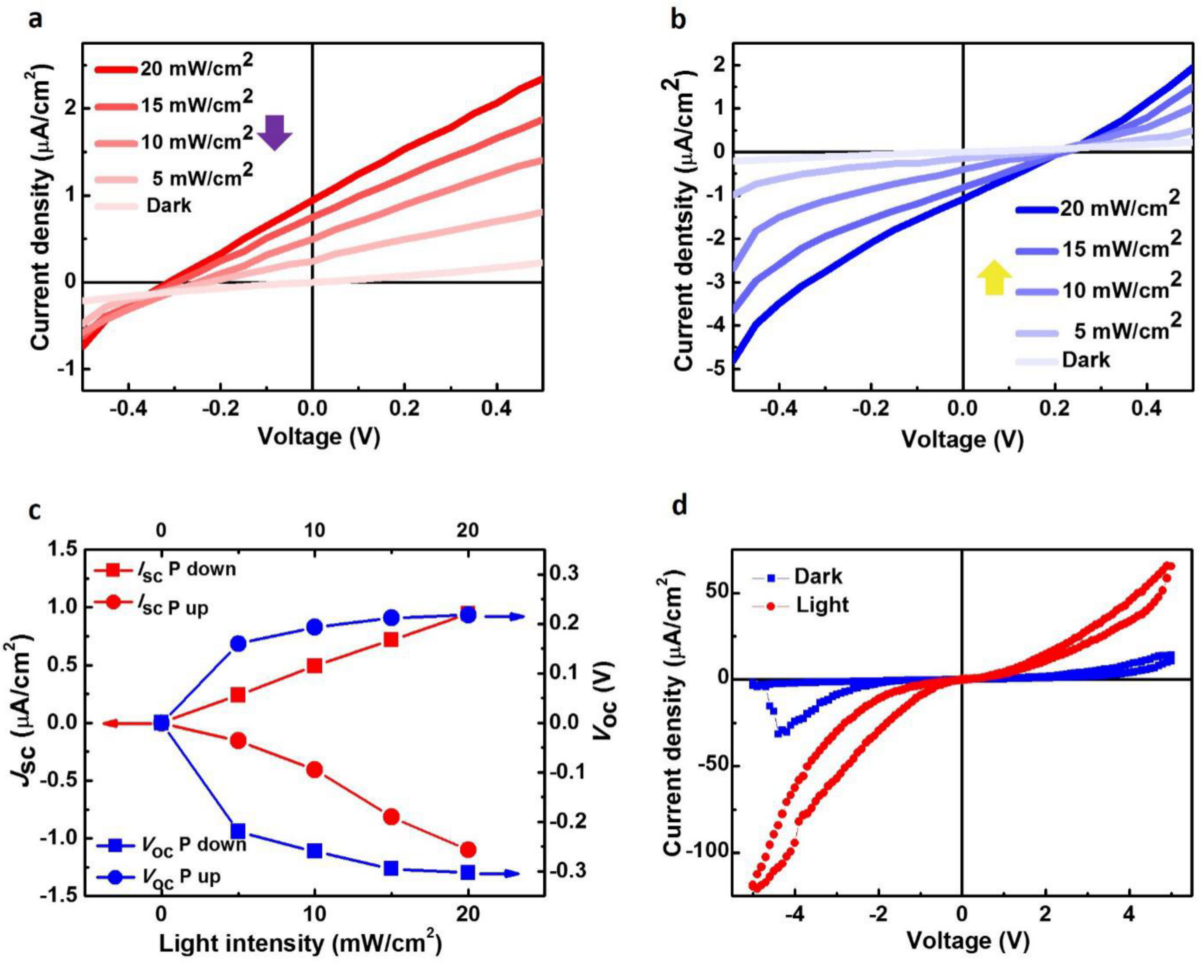

Supplementary Figure 7. Photo-response of freestanding Pt/BFO/LSMO to different light intensities. *J*-*V* curves of the flexible Pt/BFO/LSMO devices measured under different light intensities with (**a**) BFO polarization downward and (**b**) upward. Light source: halogen lamp with maximum energy density of 20 mW/cm^2. (**c**) The change of V_{oc} and J_{sc} as function of light intensity for both polarizations. (**d**) *J*-*V* switching loops of freestanding Pt/BFO/LSMO photovoltaic cells under light illumination and in dark. The purple and yellow arrows in (a) and (b) represent the downward and upward polarization of BFO.

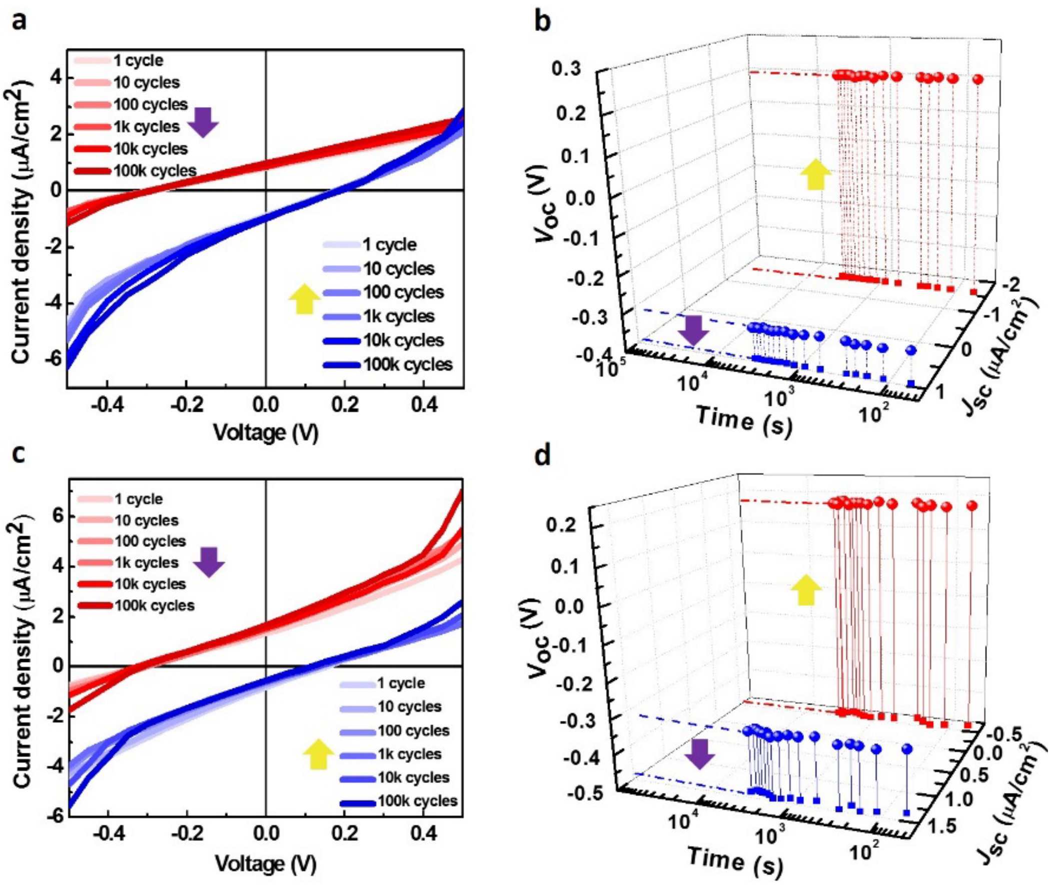

Supplementary Figure 8. Fatigue and retention results. (**a**) and (**b**) Fatigue and retention properties of the freestanding device in flat status for both polarizations. (**c**) Fatigue and (**d**) retention performances of the freestanding device in bending status for both polarizations. For the fatigue measurements, the *J*-*V* curves were measured after switching the polarization using ±3V, with the pulse width of 1ms. The freestanding BFO devices show good retention properties. Besides, the freestanding devices can sustain 100k cycles for both flat and bending status. The purple and yellow arrows represent the downward and upward polarization of BFO.

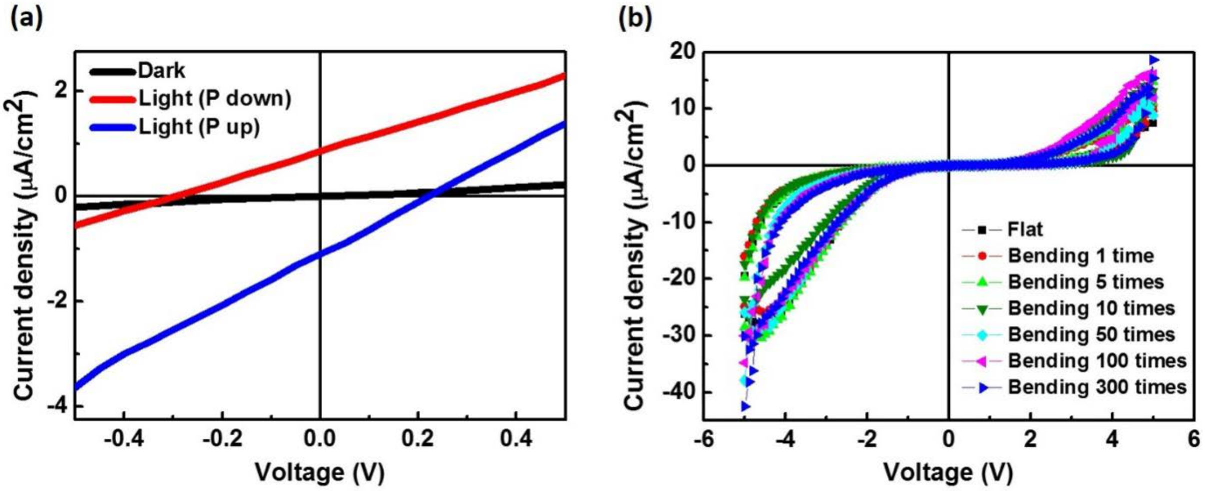

Supplementary Figure 9. Photovoltaic properties of freestanding Pt/BFO/LSMO after multiple bending. (**a**) *J-V* curves under light illumination (with light intensity of 20 mW/cm^2) and in dark for both polarizations of the flexible Pt/BFO/LSMO device after bending 300 times. (**b**) *J-V* switching loops in dark of the flexible Pt/BFO/LSMO device after bending 300 times.

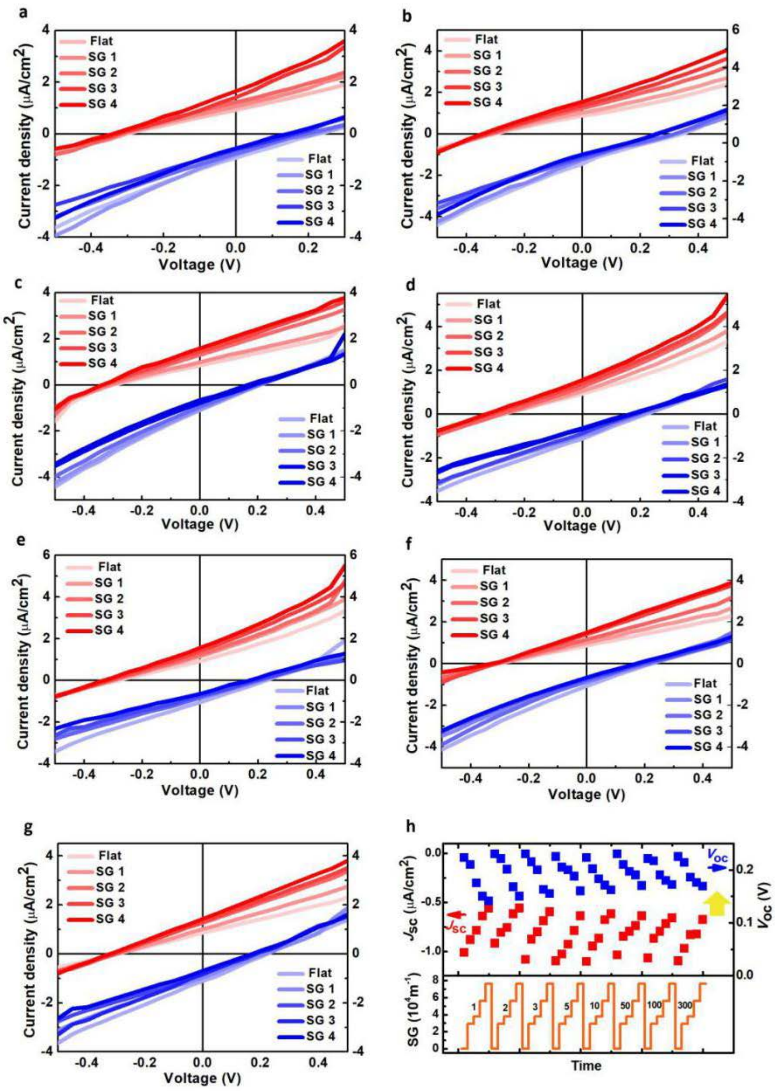

Supplementary Figure 10. Reproducibility of the tunable photovoltaic properties. (a-g) *J-V* curves under light (with intensity of 20 mW/cm^2) after 2nd, 3rd, 5th, 10th, 50th, 100th, 300th bending, respectively. After the bending, the response of the photovoltaic property to different bending radii was tested. *J-V* curve within a very small voltage range is measured to read the change of V_{oc}/J_{sc} in response to different bending radii. (**h**) The corresponding change of the V_{oc}/J_{sc} with the function of the introduced in-plane strain gradient (SG) after different bending cycles. The polarization of BFO is upward. The change of V_{oc}/J_{sc} is repeatable, which confirms the contribution of the flexoelectric effect to the photovoltaic effect of flexible BFO. SG1 to SG4 corresponds to the strain gradients of four different bending radii in Fig. 1b. The yellow arrow in (h) represents the upward polarization of BFO.

Supplementary Table 1. The calculated strain gradient and the fitted flexo-voltage (ΔV_{oc}).

Bending radius (m)	In-plane strain gradient (m^{-1})	Out-of-plane strain gradient (m^{-1})	Fitted $\Delta E_{z,flex}$ (V/m)	Fitted $\Delta V_{oc} = \Delta E_{z,flex} * t_{BFO}$ (V)
0.0036	29360.83057	-8808.24917	-202013.267	0.02021
0.0028	37749.63931	-11324.89179	-259731.344	0.02598
0.0019	55631.0474	-16689.31422	-382761.98	0.03828
0.0014	75499.27862	-22649.78359	-538900.12	0.05389

$$\varepsilon_{\text{ip}} = \frac{\frac{Y_1 {t_1}^2}{2} + Y_2 t_2 \left(t_1 + \frac{t_2}{2}\right) + Y_3 t_3 \left(t_1 + t_2 + \frac{t_3}{2}\right) + Y_4 t_4 \left(t_1 + t_2 + t_3 + \frac{t_4}{2}\right)}{r(Y_1 t_1 + Y_2 t_2 + Y_3 t_3 + Y_4 t_4)}$$

The in-plane strain ε_{ip} in BFO film can be estimated using the above equation (Equation (2) in the main text). Using these parameters (from top to bottom):

1st layer is BFO, $t_1 = t_{BFO}$ = 100 nm, $Y_1 = Y_{BFO}$ = 144 GPa[1]

2nd layer is LSMO, $t_2 = t_{LSMO}$ = 15 nm, $Y_2 = Y_{LSMO}$ = 500 GPa[2]

3rd layer is PDMS, $t_3 = t_{PDMS}$ = 5 um, $Y_3 = Y_{PDMS}$ = 3 MPa[3]

4th layer is PET, $t_4 = t_{PET}$ = 15 μm, $Y_4 = Y_{PET}$ = 3.5-11 GPa (https://www.makeitfrom.com/material-properties/Polyethylene-Terephthalate-PET-PETE. A medium value of 7.5 GPa was used here.)

The in-plane strain gradient SG_{ip} in BFO can therefore be estimated as ε_{ip}/t_{BFO}. By putting into all the values, in-plane strain gradient SG_{ip} in BFO can be obtained, which is $105.7/r$ m^{-1}. Note that, Since BFO layer is much thinner than the substrate, choosing a thinner flexible substrate can increase the minimum bending radius and therefore more tunabilities can be realized.

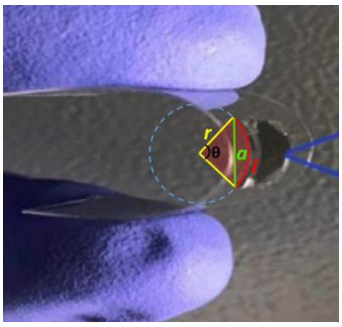

Supplementary Figure 11. Schematic of how the bending radius is calculated.

Supplementary Table 2. Values of the bending radius.

Arc length, *l* mm	Chord length, *a* mm	Calculated radius, *r* mm
4	3.8	3.6
4	3.6	2.8
4	3.3	1.9
4	2.8	1.4

By putting the value of *r* from Supplementary Table 2, in-plane strain gradient can be obtained, as shown in Supplementary Table 1, and the out-of-plane strain gradient can be obtained by taking account of the Poisson ratio of BFO (-0.3), $\nu = -\frac{\varepsilon_{\mathrm{op}}}{\varepsilon_{\mathrm{ip}}}$. The Poisson ratio for BFO film is -0.3.

As discussed in the main text, the value of λ can be back calculated, which is $\lambda = \frac{\Delta V_{\mathrm{oc}}\varepsilon_{\mathrm{o}}\, a_{\mathrm{BFO}}}{t_{\mathrm{BFO}}\, e\, SG_{\mathrm{op}}}$, (Equation (4) in the main text), where ΔV_{oc} is the flexo-photovoltage shown in Fig. 4f (P up and P down), t_{BFO} is the thickness of BFO film, and SG_{op} is the out-of-plane strain gradient in BFO. By putting the experimental data and other parameters, we can obtain the corresponding value of λ, as shown in Supplementary Table 3. Then, an average value of 0.5 for λ is obtained, with the standard deviation of ±0.17. Using the value of 0.5 for λ, the flexoelectric field and flexo-photovoltage (ΔV_{oc}) can be fitted using Equation (3) in the main text. The fitted values are listed in Supplementary Table 1.

Supplementary Table 3. The calculated value of λ corresponding to each ΔV_{oc} in Fig. 4f.

ΔV_{oc}	0.012	0.018	0.032	0.053	0.0141	0.0344	0.056	0.07
λ	0.29701	0.34651	0.41801	0.51104	0.34899	0.66222	0.73153	0.67377

Supplementary Table 4. Calculated gauge factor (GF) based on the change of the photo-resistance due to bending. ε_{ip} is the strain in freestanding BFO film corresponding to four different bending radii in the study.

ε_{ip}	0.29%	0.37%	0.55%	0.75%
$\Delta R/R_0=(R-R_0)/R_0$	0.105	0.138	0.215	0.29
GF=$\Delta R/R_0\varepsilon$	36.2	37.3	39.1	38.6

Supplementary References:

1. Dong, H., Chen, C., Wang, S., Duan, W., Li, J. Elastic properties of tetragonal $BiFeO_3$ from first-principle calculations. *Appl. Phys. Lett.* **102**, 182905 (2013).

2. Huang, Q. J., Cheng, Y., Liu, X. J., Xu, X. D., Zhang, S. Y. Study of the elastic constants in a $La_{0.6}Sr_{0.4}MnO_3$ film by means of laser-generated ultrasonic wave method. *Ultrasonics*, **44**, e1223 (2006).

3. Wang, Z., Volinsky, A. A., Gallant, N. D. Crosslinking effect on polydimethylsiloxane elastic modulus measured by custom-built compression instrument. *J. Appl. Polym. Sci.* **131**, 41050 (2014).
